# Replication Rates of *Mycobacterium tuberculosis* in Human Macrophages Do Not Correlate with Mycobacterial Antibiotic Susceptibility

**DOI:** 10.1371/journal.pone.0112426

**Published:** 2014-11-11

**Authors:** Johanna Raffetseder, Elsje Pienaar, Robert Blomgran, Daniel Eklund, Veronika Patcha Brodin, Henrik Andersson, Amanda Welin, Maria Lerm

**Affiliations:** Division of Microbiology and Molecular Medicine, Department of Clinical and Experimental Medicine, Faculty of Health Sciences, Linköping University, Linköping, SE-58185, Sweden; The Ohio State University, United States of America

## Abstract

The standard treatment of tuberculosis (TB) takes six to nine months to complete and this lengthy therapy contributes to the emergence of drug-resistant TB. TB is caused by *Mycobacterium tuberculosis* (Mtb) and the ability of this bacterium to switch to a dormant phenotype has been suggested to be responsible for the slow clearance during treatment. A recent study showed that the replication rate of a non-virulent mycobacterium, *Mycobacterium smegmatis,* did not correlate with antibiotic susceptibility. However, the question whether this observation also holds true for Mtb remains unanswered. Here, in order to mimic physiological conditions of TB infection, we established a protocol based on long-term infection of primary human macrophages, featuring Mtb replicating at different rates inside the cells. During conditions that restricted Mtb replication, the bacterial phenotype was associated with reduced acid-fastness. However, these phenotypically altered bacteria were as sensitive to isoniazid, pyrazinamide and ethambutol as intracellularly replicating Mtb. In support of the recent findings with *M. smegmatis*, we conclude that replication rates of Mtb do not correlate with antibiotic tolerance.

## Introduction

Tuberculosis (TB) is caused by *Mycobacterium tuberculosis* (Mtb), which primarily infects alveolar macrophages. Depending on the host immune status, the infection has different outcomes. In immunocompetent hosts, the bacterium may be controlled through innate immune mechanisms and/or by adaptive immunity [1], [2]. In some individuals, the immune system fails to control the infection and the disease progresses to active TB. Factors that contribute to disease progression include HIV co-infection, malnutrition and predisposing genetic variations [1].

Treatment of TB requires administration of several drugs for at least 6 to 9 months, leading to high costs, side-effects and the emergence of drug-resistant strains associated with patient non-compliance. Therefore, one of the key elements in improved global TB control is a more effective treatment regimen to shorten the time of sterilizing antibiotic therapy by several months. Altered bacterial phenotypes have been suggested to be responsible for tolerance of Mtb against antibiotics, and the prevailing view is that slow- or non-replicating bacteria in hypoxic granulomas are phenotypically tolerant towards antibiotics and thus responsible for the long time required for TB treatment. The hypoxic conditions in the granuloma have been mimicked *in vitro* by progressive oxygen depletion of cultures, rendering Mtb tolerant to isoniazid (INH) [3]. Although the absence of oxygen could directly affect the efficacy of INH [4], [5], [6], the tolerance has been attributed to the absence of replication [3].

In the human lung, Mtb can persist without the presence of granuloma [7], or in replicating and non-replicating states in subclinical lesions [8]. In the mouse model, Mtb can persist [9] although mice do not form hypoxic granulomas [10], [11], [12], and before the onset of adaptive immunity, substantial killing occurs [13]. Altogether, this speaks for a major role for innate immunity, at least during the early phase of infection and raises the question of tolerant Mtb being located outside of granulomas. Macrophages, constituting the primary target of infection and the first line of host defense, exert a range of pressures on the bacilli, forcing them to adapt to the harsh intracellular conditions and to shift phenotype, as shown earlier in different macrophage-based models [14], [15], [16] and mimicked in broth models [17]. We have shown that primary human macrophages are able to control bacterial net growth through mechanisms dependent on phagolysosomal functionality [18]. So far, Mtb replication and death rates have been difficult to determine and often neglected, although considerable evidence exists for divergent numbers of live and dead (or non-culturable) bacteria *in vivo* and *in vitro* [13], [19], [20], [21]. The link between mycobacterial replication and drug tolerance is still not clear, and a recent study in a non-virulent mycobacterium, *Mycobacterium smegmatis*, elegantly showed that tolerance correlates with expression fluctuations of katG (a mycobacterial catalase-peroxidase which protects Mtb from oxidative stress but also transforms INH into its active form [22]) and is independent of replication rate [23]. Furthermore, asymmetrical division of *M*. *smegmatis* resulting in phenotypically heterogeneous siblings growing at different rates did not cause any differences in antibiotic susceptibilities [24].

With this study, we take these findings into a more physiological setting and evaluate drug susceptibility of phenotypically different, virulent Mtb inside human monocyte-derived macrophages (hMDM). We observed that hMDMs are able to restrict intracellular Mtb net growth for at least 10 days, provided that the initial bacterial burden was low. During growth restriction, Mtb displayed a phenotype that was rich in lipid bodies, but negative for acid-fast staining, both of which are features that have been linked to persistent Mtb. A higher bacterial burden, on the other hand, promoted an actively replicating phenotype that was positive for acid-fast staining. Finally, we tested whether the susceptibility towards first- and second-line TB drugs was different in the characterized phenotypes. Consistently with the findings obtained with *M. smegmatis*, we demonstrate that an altered replication rate of Mtb did not influence the susceptibility of the bacterium to antibiotics.

## Results

### Macrophages control Mtb net growth during a low burden infection

**Figure 1:**
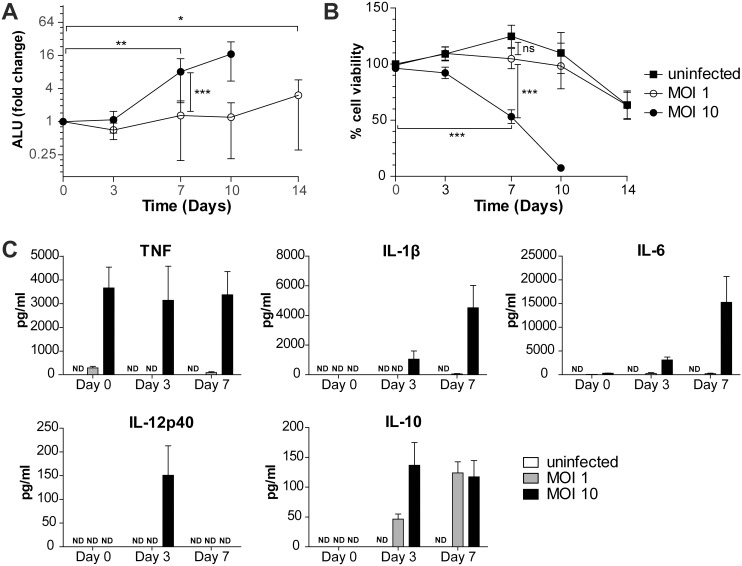
Kinetics of Mtb growth, macrophage cell death and cytokine secretion. Bacterial fold change (A; normalized to day 0 values of the respective MOI) and percentage of macrophage survival (B; normalized to uninfected controls on day 0) were measured during 14 days of H37Rv infection using luminometry for bacterial numbers and calcein-AM for macrophage viability. Arbitrary luminescence units (ALU) for medium supernatant and lysate (Figure S1) measurements were added to give totals (A). n = 7–32 and symbols and error bars represent means and 95% confidence intervals. Comparisons between MOI 1 and MOI 10 (and uninfected controls for (B)) at different time points were done using unmatched 2-way ANOVA of normalized values and Bonferroni post-hoc test for multiple comparisons. Significant changes compared to day 0 were determined using 1-way ANOVA of normalized values and Dunnett’s test, and only the first time point significantly different from day 0 is indicated with asterisks (A and B). *p<0.05, **p<0.01, ***p<0.001. (C) For cytokine analysis, medium supernatants were saved on the respective days of infection and analyzed by cytokine bead array for the indicated cytokines. n = 5–7 and bars and error bars depict means and SEMs, respectively. ND: Not detected.

In order to establish whether unstimulated hMDMs were able to restrict growth of virulent Mtb for an extended period of time, we performed infection experiments through 14 days of infection. We found that infection of hMDMs with Mtb H37Rv at a multiplicity of infection (MOI) of 1 did not result in any significant net increase in bacterial numbers for at least 10 days, a period during which cell viability of infected cells was similar to uninfected cells (Figure 1A and B). On the contrary, infection with a higher MOI (MOI 10) resulted in significant bacterial growth by day 7 (Figure 1A), coinciding with extensive cell death (Figure 1B) and release of Mtb from dying cells causing an increase in the extracellular fraction, but not in the cell-associated fraction (Figure S1).

The different outcomes of MOI 1 and MOI 10 infection prompted us to map the inflammatory response of the cells to the different bacterial loads. Cells infected with MOI 10 released high amounts of TNF at day 0, and of IL-1β, IL-6, IL-12p40 and IL-10 starting from day 3. Cells infected with MOI 1 initially secreted TNF at levels corresponding to approximately 10% of the amount secreted from the MOI 10-infected cells. However, at day 3, there was no detectible TNF secretion from MOI 1-infected cells, followed by a slight increase by day 7. The other investigated pro-inflammatory cytokines were low (IL-6 on day 3 and IL-1β and IL-6 on day 7) or undetectable (IL-1β and IL-12p40 on day 3) during MOI 1 infection. On the other hand, the levels of the anti-inflammatory cytokine IL-10 increased by day 3 and were equal for both MOIs by day 7. Uninfected cells did not release any of the cytokines measured (Figure 1C), and hMDMs exhibited a heterogeneous phenotype at the time of infection, expressing both M1 and M2 macrophage makers (Figure S2 and Table S1), corresponding to a more dynamic classification of macrophages, as proposed by Mosser and Edwards [25], rather than the conventional IFNγ-/IL-4-induced M1/M2 phenotypes.

During the course of infection, bacterial numbers were measured using a H37Rv strain carrying a luciferase-encoding plasmid with a hygromycin resistance marker (pSMT1). To rule out the possibility of changes in luciferase expression after infection, we routinely correlated arbitrary luminescence units (ALU) to bacterial CFU. During extended macrophage infection, ALU and CFU correlated well and most importantly, bacterial numbers were not underestimated when using luminometry (Figure S3A). Furthermore, the luminescent signal did not diminish when hygromycin is absent indicating that plasmid loss does not occur during a time period of at least 14 days (Figure S3B). This is further supported by earlier publications on the same plasmid showing that CFU and ALU correlate well for at least 60 days in a murine infection model [26].

### Bacterial replication rates are dependent on the initial bacterial burden

To investigate whether the absence of intracellular net growth during MOI 1 infection reflects bona fide non-replicating bacteria or a dynamic equilibrium (growth balanced by killing by macrophages), we used the replication clock plasmid [13]. Briefly, this low copy plasmid is lost from each generation at a constant rate, and together with the proportion of plasmid-containing Mtb, this rate can be used to derive the replication (r) and death (d) rates of the bacteria in a given population at a given time point.

**Figure 2:**
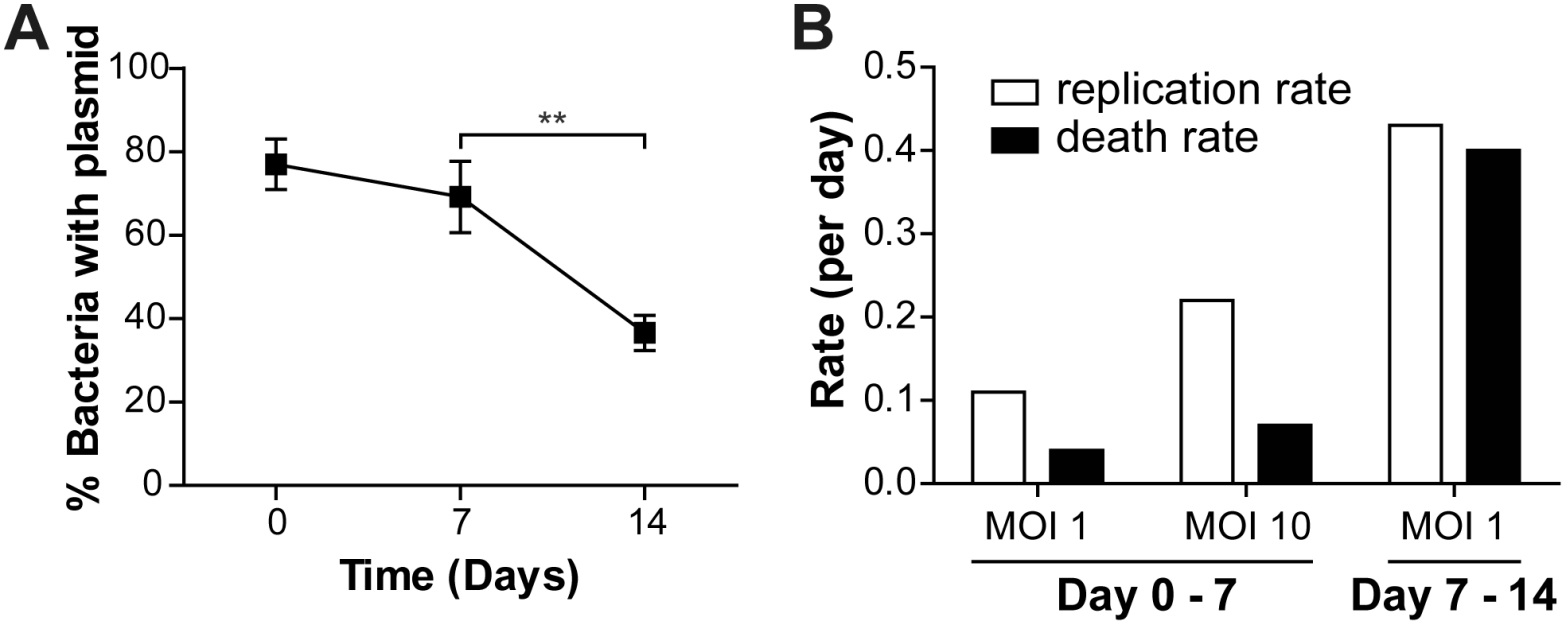
Loss of clock plasmid in the intracellular fraction and estimated mycobacterial replication and death rates. (A) CFU counts from cell lysates during MOI 1 infection on kanamycin-containing plates normalized to total CFU counts on plates without kanamycin. Differences in percentage of bacteria containing the clock plasmid was analyzed using 1-way ANOVA and Tukey’s post-hoc test. n = 8–11. **p<0.01. (B) Estimated replication and death rates (per day) for intracellular Mtb were calculated from clock plasmid CFU data. Rates for MOI 10 infection between day 7 and 14 could not be determined due to extensive cell death.

Analysis of plasmid loss from intracellular bacteria revealed that during the initial phase of MOI 1 infection, there was no significant loss of plasmid (Figure 2A, estimated generation time of 6.5 days or 158 h). Between day 7 and 14 of MOI 1 infection, a shorter generation time of 1.5 days (38 h) was accompanied by an increase in bacterial death rate (r = 0.43 and d = 0.40, Figure 2B), suggesting growth balanced by killing during the later phase of infection. Both phases are consistent with the absence of net growth as observed in Figure 1A. For the time span between day 0 and day 7 during MOI 10 infection, Mtb was estimated to replicate once every 3 days (76 h, r = 0.22, d = 0.07, Figure 2B). The larger difference between r and d during MOI 10 compared to MOI 1 infection is reflected in the observed net growth during MOI 10 infection (Figure 1A). The method likely underestimates the replication rate (and hence overestimates the generation time) of the MOI 10 infection, since dying macrophages release replicating bacteria into the supernatant. For comparison, we determined the generation time in broth for H37Rv to be 37 hours.

### Intracellular bacterial phenotype

**Figure 3:**
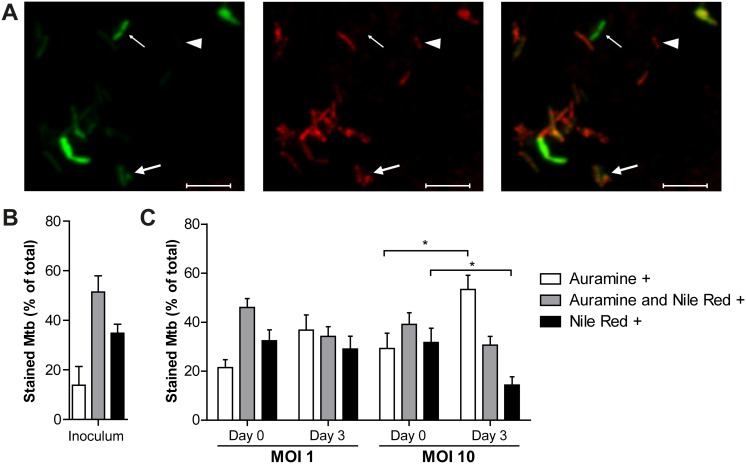
Phenotypic characteristics of Mtb inoculum and during macrophage infection. (A) Representative image of intracellular Mtb stained with Auramine O and Nile Red, counted as either Auramine O-positive (thin arrow), Nile Red-positive (arrowhead) or as positive for both stainings (thick arrow). Scale bar: 5 µm. (B) Percentage of bacteria stained with Auramine O, Nile Red or both in the inoculum. n = 3. (C) Percentage of Auramine O/Nile Red-positive intracellular bacteria, using hMDMs from 20 different donors. Significant changes were determined using 1-way ANOVA comparison followed by Bonferroni’s multiple comparison test. *p<0.05. Bars and error bars represent means and SEMs respectively.

Next, we characterized whether the slow-growing Mtb during MOI 1 infection displayed an altered phenotype, as compared to the actively growing Mtb during MOI 10 infection. Persistent Mtb are characterized by reduced acid-fastness and intrabacterial accumulation of lipid bodies, as observed *in vivo* in the lungs of latently infected individuals, in sputum from TB patients and in an *in vitro* multiple-stress dormancy model [17], [27], [28]. In order to determine the phenotype of intracellular Mtb, we implemented a combined acid-fast (Auramine O) and lipid body (Nile Red) staining technique [17]. Representative images of stained intracellular Mtb are shown in Figure 3A. The inoculum displayed a mixed phenotype, with 14% of bacteria being positive for Auramine O only, 51% positive for both Auramine and Nile Red, and 35% positive for Nile Red only (Figure 3B).

One hour after infection (Day 0) with either MOI 1 or MOI 10, the staining pattern of intracellular Mtb resembled the inoculum (Figure 3B and C), indicating that no phenotypic shift occurred during the first hour of infection. This phenotype was not altered during MOI 1 infection by day 3 (Figure 3C), suggesting that the macrophages were able to maintain the initial bacterial phenotype. In contrast, there was a shift in the staining pattern of Mtb infecting the macrophages at the MOI 10 ratio, with a significant increase in Auramine-positive and a significant decrease in Nile Red-positive bacteria (Figure 3C). This phenotypic shift coincided with the bacterial replication observed during MOI 10 infection.

### Antibiotic susceptibility of different Mtb phenotypes

Having established that our primary human macrophages were able to maintain an altered, slow-growing and lipid-rich phenotype of Mtb, we tested whether the sensitivity of these bacteria towards some first- and second-line antimycobacterial drugs was different from the sensitivity of actively replicating, acid-fast bacteria in the same system.

**Figure 4:**
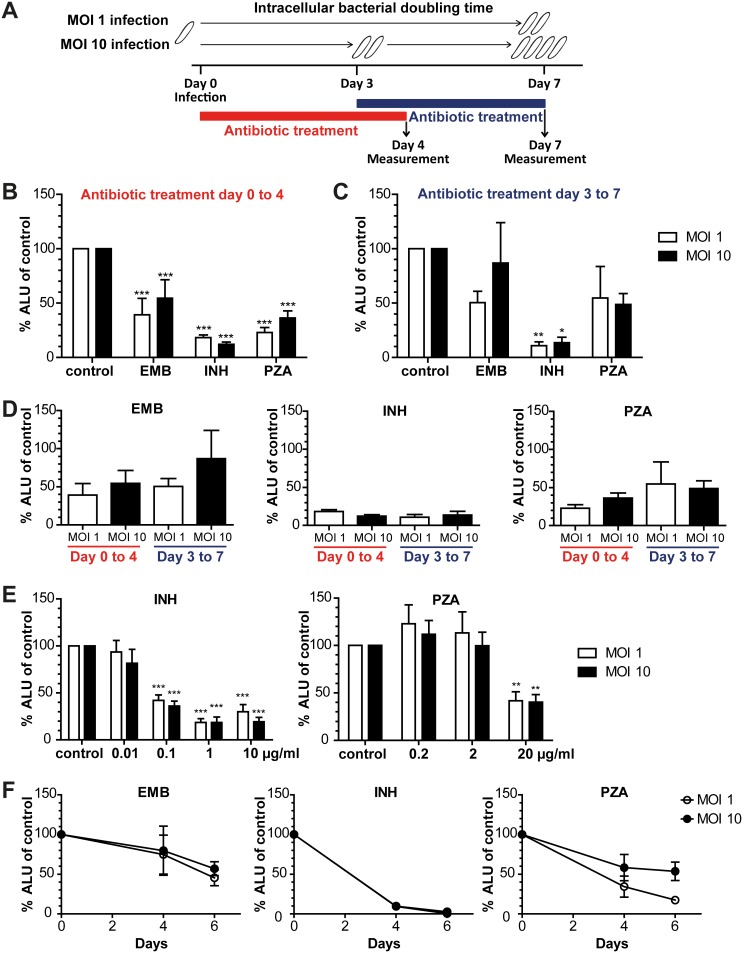
Antibiotic susceptibility of intracellular Mtb. (A) Schematic outline of the experiments, with antibiotics being added either 1 h (B) after infection or after 3 days (C) when Mtb in MOI 10-infected cells already had replicated once. Intracellular bacteria were quantified 4 days later on day 4 or 7, respectively. Antibiotics were used at the following concentrations derived from human peak serum levels: 1 µg/ml ethambutol (EMB), 10 µg/ml isoniazid (INH) and 20 µg/ml pyrazinamide (PZA). ALU as a measure of bacterial numbers were normalized against untreated controls of the same donor. Significant changes were determined using 2-way ANOVA followed by Bonferroni’s post-hoc test comparing treated samples to untreated control. Differences between MOIs were not significant. (D) contains the data from (B) and (C). Groups were compared using 1-way ANOVA and Tukey’s post-hoc test. No significant differences were found. (E) Lower concentrations of INH and PZA were added than in B–D (0.01, 0.1 and 1 µg/ml INH and 0.2 and 2 µg/ml PZA), and intracellular bacteria were measured on day 4. Significant differences between treated samples and untreated control were determined using 2-way ANOVA followed by Bonferroni’s post-hoc test (indicated with asterisks). No significant difference between MOIs was found. (F) Antibiotics were added 1 h after infection as in (B) and (E), but infection was extended beyond day 4 with another measurement on day 6. n = 5–10 in (B–D), n = 3 in (E) and n = 3 in (F). Bars and error bars represent means and SEMs, respectively. *p<0.05, **p<0.01 and ***p<0.001.

To this end, antibiotics at concentrations based on human peak serum levels were added 1 hour after infection with either MOI 1 or MOI 10 and the number of intracellular bacteria was measured 4 days later. At this time point, no replication had taken place in the MOI 1 situation, whereas one replication had occurred in the MOI 10 situation (as determined by the clock plasmid experiment), thus reflecting situations with non-replicating and replicating bacteria, respectively (schematically outlined in Figure 4A). Significant reduction of bacterial numbers was seen after treatment with three of the first-line drugs ethambutol (EMB), INH and pyrazinamide (PZA) (Figure 4B), but there was no difference between the two MOIs. One possible interpretation of this result may be that the bacteria need time to shift to a different phenotype in the MOI 10 situation. To test this, we performed an additional experiment, in which the antibiotics were added 3 days after infection. Again, bacterial numbers were determined 4 days after addition of antibiotics, and INH was found to significantly kill intracellular bacteria (Figure 4C), but without any difference in antibiotic susceptibility between MOI 1 and MOI 10 infection. The same set of experiments was carried out with second-line drugs (amikacin, capreomycin, kanamycin, metronidazole and streptomycin), but none of the tested drugs caused any significant reduction in bacterial numbers as compared to untreated controls. As observed with the first-line drugs, there was no difference between the two MOIs (Figure S4), and none of the tested first- and second-line drugs rescued cell viability as compared to the untreated controls (Figure S5).

Using the data presented in Figure 4B and 4C, we made a statistical comparison of the percentage of bacteria remaining after 4 days of antibiotics treatment independently of whether the antibiotics were added at day 0 or day 3. The efficacy of EMB, INH and PZA did not differ between MOIs and time points (Figure 4D), indicating that the antibiotic susceptibility was not dependent on the replicative state of the bacteria. In order to rule out that the concentrations of drugs are too high to discriminate between tolerant and susceptible bacteria, we tested lower concentrations of the drugs with the best intracellular effect, INH and PZA, but again, no significant differences between MOIs could be observed (Figure 4E). Treatment with antibiotics was also extended beyond 4 days, showing that bacterial numbers can be further diminished (Figure 4F), which speaks against a residual tolerant population.

The antibiotics were demonstrated to be effective against H37Rv in 7H9 broth cultures (Figure S6), with the exception of PZA that requires acidic pH for activity and was not expected to have any effect in broth, as well as metronidazole which requires anaerobic conditions [3], [29]. All the other first and second line drugs effectively killed bacteria in broth, indicating that the bacteria used are genotypically susceptible to those antibiotics.

We also assessed whether treatment with INH, PZA and EMB affected the two studied phenotypes differently using the Auramine O/Nile Red staining protocol on intracellular Mtb after 4 days of infection. The activities of the studied drugs did not affect a certain phenotype more than the other, however, the reliability of the method could have been influenced by the fact that also antibiotic-killed bacteria were stained as indicated by the fragmented appearance of many bacteria (not shown).

## Discussion

Aiming to understand how Mtb phenotypes relate to the lengthy treatment required for TB, we investigated antibiotic susceptibility of Mtb inside macrophages. Two major findings guided the investigation: first, evidence has accumulated that not only necrotic granulomas but also macrophages can harbor altered phenotypes of Mtb [14], [15], [16], [30], [31], [32], and second, that antibiotic susceptibility might not necessarily be coupled to mycobacterial replication rate [23].

We found that unstimulated primary human macrophages harbored an altered phenotype of Mtb during low-burden infection, characterized by slow replication, lipid bodies and reduced acid-fast staining and that this phenotype exhibited similar antibiotic susceptibility as did actively replicating, acid-fast Mtb. The control of bacterial net growth in human macrophages infected with a low bacterial burden was earlier shown to depend on effective phagosomal acidification [18], while macrophages infected with Mtb at higher MOIs undergo necrotic cell death coinciding with intracellular replication [33]. In the present study, long-term infection experiments showed that the balance between macrophages and Mtb at the low MOI could be maintained for at least 10 days. While restriction of mycobacterial growth has been described in other macrophage-based systems, this was dependent on manipulation of the macrophages via factors such as IFN-γ, TNF, GM-CSF or hypoxic conditions [14], [15], [16]. These studies only describe the absolute numbers of intracellular bacteria and do not provide information about bacterial replication and killing rates. We included the clock plasmid replication rate analysis [13] in order to distinguish lack of replication from coincident replication and death, both of which would result in unchanged bacterial numbers over time. During low MOI infection, an early phase of slow replication was followed by a phase of faster replication and compensatory killing. The fact that a period of bacterial turnover follows the initial phase suggests that Mtb dynamically cycles between actively replicating and non- or slow-replicating states. A possible explanation for the low bacterial death rate during the early stage of MOI 1 infection is that macrophage effector functions are ineffective against this phenotype of Mtb, which would provide a rationale for its existence. Our observation of an initial stage of slow-replicating bacteria is contrary to previous findings with Mtb CDC1551 infection of murine bone marrow-derived macrophages [19], which showed higher replication (and death) rates associated with a net decrease in bacterial load in the initial phase of the infection, followed by lower replication (and death) rates coincident with a net increase in bacterial numbers. The divergent results may be explained by differential inherent ability of murine and human macrophages to control Mtb infection, by strain variability and possibly also by factors affecting the phenotype(s) of the Mtb inoculum.

The absence of cytokine release from uninfected cells confirms that the cells were not pre-activated and suggests that factors acting inside the cells rather than mediators acting via auto- or paracrine routes contribute to the restriction of intracellular Mtb growth. Analyzing the Mtb inoculum, we found that both replicating (acid-fast-positive) and persister-like (lipid-rich/acid-fast-negative) bacteria were present, probably due to our unagitated Mtb culture conditions. This inoculum phenotypically resembles Mtb found in sputum from TB patients [28], thus constituting a physiologically relevant source of Mtb. Characterization of intracellular Mtb at the higher MOI revealed a significant shift towards the acid-fast-positive, lipid body-negative phenotype, which correlated with a higher replication rate. In contrast, the mixed Mtb phenotypes observed during initial infection were maintained throughout the experiment at the low MOI. Cell wall alterations leading to decreased acid-fastness are features of Mtb persistence *in vivo* [27], [34], and both lost acid-fastness and accumulation of lipid bodies can be induced in a multiple-stress dormancy model [17], and in hypoxic macrophages [14]. In contrast, we show that unstimulated macrophages can harbor an altered Mtb phenotype under normal oxygen pressure. We were unable to quantify whether simultaneous bacterial replication and persistence occurs within the same cell since we could not distinguish the borders of individual cells using this staining protocol. However, the frequent appearance of Auramine O-positive bacteria in the vicinity of Nile Red-positive bacilli suggests that both phenotypes can exist in the same cell. Since the inoculum used in this study contained a mixture of Mtb phenotypes, we cannot make conclusions regarding the ability of the macrophages to induce a phenotypic shift from actively replicating to a lipid-body-rich and acid-fast-negative phenotype. Although this question needs further attention, previous studies have reported induction of stress-regulated genes in Mtb upon uptake into macrophages [19], [35], suggesting that the pathogen alters its phenotype to endure the stressful intracellular environment.

Phenotypic drug tolerance has been attributed to the absence of replication, e.g. in *E. coli* [36] and recently also in intracellular *Salmonella* [37]. We found that EMB, INH and PZA efficiently killed intracellular bacilli, and the extent of killing was independent of the MOI, i.e. of the bacterial replication rate. Regardless of MOI and of the time point of addition of antibiotics, the susceptibility pattern was similar, suggesting that antibiotic tolerance of intracellular bacteria does not correlate with bacterial replication rates. Our results provide two possible explanations to the enigmatic fact that INH, which has been traditionally viewed as a drug that is ineffective against non-replicating Mtb [3], is successfully used to treat latent TB [38]. First, we show that it is possible that a macrophage population can balance growth by killing, housing actively replicating bacteria without a net increase of bacterial load, which has been shown to be the case in a mouse model for chronic TB [13]. More importantly, we show that independently of replication rates, INH is as effective in killing Mtb. Our data are supported by the recent study by Wakamoto et al. [23], in which INH tolerance of *Mycobacterium smegmatis* correlates with fluctuations in *katG* expression rather than replication rate. Another study led to a different conclusion and showed THP1 macrophage-induced tolerance to INH in replicating *Mycobacterium marinum* bacteria [30]. In the present study, we cannot exclude the possibility that activated or immunosuppressed macrophages would have rendered Mtb tolerant to INH or the other drugs tested here. We did not measure KatG fluctuations, and to our knowledge, this has not been studied inside macrophages. Furthermore, our model does not necessarily account for the Mtb phenotype found in the hypoxic core of granulomas, where Mtb might undergo a truly non-replicating state (Wayne) or be tolerant to INH due to other factors like oxygen inavailability [4], [5]. The absence of activity of second-line drugs in our study is most likely explained by limited intracellular activity of these drugs [39]. Other studies, showing good intracellular effect of these antibiotics, did not investigate macrophage viability [40].

Although first-line drugs effectively killed the bacteria in MOI 10-infected cells in the present study, the treatment did not significantly rescue macrophage viability. The finding suggests that the initial bacterial load rather than the absolute numbers of bacteria determines cell death, however, the reason for this needs further investigation.

To conclude, unstimulated human macrophages were able to maintain phenotypically altered Mtb exhibiting some characteristics of persisters, which supports a role for innate immune cells in latent TB. Being based on infected primary human macrophages as opposed to broth cultures, our model provides a physiological environment in which altered Mtb phenotypes can be studied. Furthermore, we challenge the view that Mtb replication rates determine antibiotic susceptibility inside macrophages.

## Materials and Methods

### Ethics statement

Blood, collected at the blood bank at Linköping University Hospital, was obtained from healthy donors, who had given written consent for research use of the donated blood in accordance with the Declaration of Helsinki. Since blood donation is classified as a negligible risk to the donors and since only de-identified samples were delivered to the researchers, this study did not require a specific ethical approval according to paragraph 4 of the Swedish law (2003∶460) on Ethical Conduct in Human Research.

### Bacteria

Mtb H37Rv (ATCC) carrying the luciferase-encoding pSMT1 plasmid [26] or both the pSMT1 and the “clock plasmid” pBP10 [13] were grown in Middlebrook 7H9 broth (Difco, Becton Dickinson) supplemented with glycerol, Tween-80 and albumin-dextrose-catalase (ADC, Becton Dickinson) as described earlier [41], and reseeded into fresh medium 7 days before infection. Bacteria carrying the plasmids were selected with 100 µg/ml hygromycin (Sigma) for pSMT1 and 75 µg/ml kanamycin (Sigma) for pBP10.

### Human monocyte-derived macrophages

For the preparation of hMDMs from heparinized whole blood or buffy coats, isolation of the mononuclear cell fraction using LymphoPrep (Axis Shield) and differentiation of monocytes were performed as described [18], [33]. Monocytes were allowed to differentiate into hMDMs for 5–8 days in Dulbecco’s Modified Eagle Medium (DMEM, Gibco) containing 80 µM L-Glutamine (Gibco) and 10% non-heat inactivated human serum (from blood bank at Linköping University Hospital) pooled from 5 donors. The day before infection, cells were trypsinized and re-seeded in serum-containing medium: 1×10^5^ cells/well in triplicates in black 96-well plates (Greiner) for determination of bacterial growth and cell viability, and 2.5×10^5^ cells/coverslip for staining.

### Macrophage characterization

For staining of intracellular macrophage markers, cells were treated with Cytofix/Cytoperm™ (BD Pharmingen) before staining with antibodies. Antibody manufacturers and concentrations used are given in Table S2. Samples stained with fluorophore-conjugated secondary antibodies only served as background controls for intracellularly stained samples. Isotype antibody-treated cells were used as background controls and single- and non-stained cells for color compensation. 10,000 events/sample were acquired using a Gallios Flow Cytometer (Beckman Coulter) and data was analyzed using Kaluza or Flowjo.

### Experimental infection

For infection, bacteria were passaged through a 27 gauge needle to remove aggregates and diluted in serum-free medium as described earlier [41], then added to the macrophages at an MOI of 1 or 10. After 1 hour of incubation, the medium was replaced by fresh DMEM containing human serum. For long-term infections, medium was changed on day 3, 7 and 10. For antibiotic susceptibility experiments, antibiotics (all from Sigma Aldrich) were added 1 hour or 3 days after infection. Intracellular bacterial numbers and cell viability were evaluated 4 days after addition of antibiotics as described below. Uninfected and untreated controls were included for all time points. Day 0 measurements were done 2 to 4 hours after infection.

### Antibiotic susceptibility in broth

Mtb H37Rv expressing luciferase from the pSMT1 plasmid were prepared from the same culture as used for infection and diluted in Middlebrook 7H9 broth supplemented with Tween 80 and ADC, with or without antibiotics, to a concentration of 10^5 ^CFU/ml. Antibiotic concentrations used were the same as in the macrophage experiments. After 4 days of incubation, bacterial numbers were determined using the luminescence-based method described below.

### Measurement of bacterial numbers and cell viability

Bacterial numbers were determined by a luminescence-based method published previously [41]. Aliquots of medium supernatants and lysates containing luciferase-expressing bacteria were transferred to white 96-well plates (Greiner), and flash luminescence after injection of the luciferase substrate (1% decanal, Sigma Aldrich) was measured in a plate reader (GloMax-Multi+ Detection System with Instinct Software, Promega). The remaining supernatants were pooled, spun down and frozen at −80°C for cytokine analysis, and cell viability was determined as described below prior to subjecting the cells to hypotonic lysis. Arbitrary luminescence units (ALU) obtained from supernatant and lysate measurements were corrected for background luminescence using ALU values from uninfected cells. In order to calculate the total values for each well (intracellular and extracellular bacteria), the ALUs of the supernatant and lysates were standardized for dilutions and summed up. For bacterial growth, the median value of each triplicate of all time points was normalized to the day 0 median of the same experiment (fold change) or normalized to medians of untreated controls of the same day in the antibiotics experiments.

To determine cell viability, cells were washed three times with PBS, followed by 30 min incubation with 4 µM calcein-AM (Molecular Probes). Fluorescence was measured in a plate reader. Arbitrary fluorescence units of infected samples were normalized to those of uninfected cells measured on day 0.

### Correlating arbitrary luminescence units to CFU

In order to ensure the stable expression of the luciferase-encoding pSMT1 plasmid in Mtb H37Rv after macrophage infection and to exclude the possibility of underestimating the actual bacterial load due to plasmid loss, ALU measured in medium supernatants and lysates were repeatedly correlated to CFU obtained by traditional plating of the same samples. To do so, supernatant and lysate samples from triplicate wells were pooled, serially diluted and plated in triplicates on Middlebrook 7H10 agar supplemented with ADC. CFUs were counted after two and three weeks of incubation at 37°C, ALU und CFU calculated per well and the median CFU value was correlated to the mean ALU value (since triplicates had been pooled). In order to check for plasmid loss when bacteria are maintained without hygromycin, H37Rv expressing luciferase were grown in 7H9 broth supplemented with ADC in the presence and absence of the selecting antibiotic hygromycin. Every few days, ALUs were measured.

### Cytokine analysis

Cytokine analysis was performed using the human flex sets for TNF, IL-1β, IL-6, IL-10 and IL-12p40 for Cytokine Bead Array (Becton Dickinson), according to the manufacturer’s instructions followed by an additional fixation step (4% paraformaldehyde for 30 min). Samples were measured using a Gallios Flow Cytometer (Beckman Coulter) and data were analyzed using Kaluza software (Beckman Coulter).

### Evaluation of replication rates

The loss of the clock plasmid from intracellularly replicating bacteria was determined by CFU plating of cell lysates on Middlebrook 7H10 plates supplemented with ADC with and without 75 µg/ml kanamycin. Bacteria containing the plasmid grow on both plates, whereas CFUs of bacteria without the plasmid appear only on kanamycin-free plates. The rate of plasmid loss (segregation rate) was determined in logarithmic phase cultures to be 0.2. Bacterial replication and death rates can be calculated from the segregation rate, total CFU and plasmid containing fractions as outlined elsewhere [13].

### Staining of Mtb

Staining of the inoculum and intracellular Mtb was adapted from Garton et al. [42]. Inoculum was streaked on microscope slides, dried and heat-fixed. hMDMs infected on glass coverslips were fixed with 4% paraformaldehyde either 1 hour or 3 days after infection. Microscope slides and coverslips were treated with Auramine O solution (TB Auramine M by Becton Dickinson), acid alcohol and Nile Red (Sigma Aldrich). Between all steps, slides were washed with water. Samples were mounted with fluorescence mounting medium (DAKO). Microscopy was performed using a Zeiss LSM 700 confocal microscope, taking Z-stacks and using the Zen software (Zeiss) for image projection. Bacteria were evaluated for staining with Auramine O and Nile Red.

## Supporting Information

Figure S1 Kinetics of Mtb growth in the extracellular and cell-associated fraction. Bacterial fold-change in the macrophage supernatant (A) and lysate (B) during the long-term infection experiments shown in Figure 1A and 1B. Bacterial numbers were measured using luminometry and expressed ALU normalized to Day 0 values. n = 7–32 and symbols and error bars represent means and 95% confidence intervals. Comparisons between MOI 1 and MOI 10 at different time points were done using unmatched 2-way ANOVA of normalized values and Bonferroni post-hoc test for multiple comparisons. Significant changes compared to day 0 were determined using 1-way ANOVA of normalized values and Dunnett’s test, and only the first time point significantly different from day 0 is indicated with asterisks. **p<0.01, ***p<0.001.
(EPS)

Figure S2 Macrophage characterization. Surface (CD206, CD 163, DC-SIGN, CD86 and CD14) and intracellular (iNOS2, arginase I, and CD119) staining of hMDMs differentiated for 8 days. Plots show representative expression in one of six donors. Dashed lines show background fluorescence.
(EPS)

Figure S3 Correlation of arbitrary luminescence units to CFU, and plasmid stability. (A) ALUs from Mtb expressing luciferase were measured in aliquots of the cell lysates, and aliquots of the same samples were used for CFU plating. ALU/well and CFU/well are shown over time from one representative donor of four. (B) Mtb expressing luciferase were grown in the presence and absence of the selecting antibiotic hygromycin and bacterial numbers were quantified by luminometry. One representative experiment of two is shown.
(EPS)

Figure S4 Intracellular susceptibility of Mtb to second-line TB drugs. Antibiotics were added either 1 h after infection (A) or on day 3 (B) after infection. Intracellular bacterial numbers were measured 4 days later, on day 3 or day 7, respectively. Antibiotics were used at the following concentrations: 1 µg/ml amikacin (AMI), 30 µg/ml capreomycin (CAP), 10 µg/ml kanamycin (KAN), 10 µg/ml metronidazole (MTZ) and 10 µg/ml streptomycin (STR). Bacterial numbers were normalized against untreated controls of the same donor. Significant differences were determined using 2-way ANOVA followed by Bonferroni’s multiple comparison test comparing treated samples to untreated control. n = 3–6 and bars and error bars represent means and SEMs, respectively.
(EPS)

Figure S5 Cell viability of infected macrophages treated with first- and second-line TB drugs. First-line drug treatments in (A) and (B) correspond to the bacterial growth data shown in Figure 4B and 4C, and second-line drug treatments in (C) and (D) correspond to Figure S4. Antibiotics were added 1 h after infection (A) and (C) or on day 3 (B) and (D), and cell viability was measured at the same time point as intracellular bacterial numbers were determined, on day 4 or 7, respectively and normalized against the cell viability of uninfected cells from the same day. Significant differences were determined using 2-way ANOVA followed by Bonferroni’s multiple comparison test comparing treated samples to untreated but infected control. Bars and error bars represent means and SEMs, respectively. *p<0.05, **p<0.01 and ***p<0.001.
(EPS)

Figure S6 Antibiotic susceptibility of H37Rv in 7H9 broth. Luciferase-expressing Mtb H37Rv were inoculated in 7H9 broth and exposed to first- and second-line drugs or left untreated (Control) for 4 days. The antibiotic concentrations used were the same as in Figure 4 and Figure S4. The number of bacteria in the samples was then assessed using luminometry and normalized to untreated controls. Bars depict means from four (EMB, INH, PZA) or two (AMI, CAP, KAN, MTZ, STR) independent experiments and error bars represent SEM.
(EPS)

Table S1 Macrophage markers on hMDMs from cells from six independent donors.
(DOC)

Table S2 Antibodies used for macrophage characterization.
(DOC)
