# Supplementary material for: Replication Rates of Mycobacterium tuberculosis in Human Macrophages Do Not Correlate with Mycobacterial Antibiotic Susceptibility
Source: PLoS One. 2014 Nov 11;9(11):e112426. doi: 10.1371/journal.pone.0112426 (PMC4227709; doi:10.1371/journal.pone.0112426)
Supplement: Table S1 — Macrophage markers on hMDMs from cells from six independent donors. (DOC) [file pone.0112426.s007.doc]

**Table S1: Macrophage markers on hMDMs from cells from six independent donors**

|  |  | % positive ± SEM  (range) | MFI increase above background ± SEM |
| --- | --- | --- | --- |
| Surface markers | CD86 | 99.16 ± 0.59  (96-100%) | 105861.8 ± 11196.5 |
|  | CD206 | 16.56 ± 3.12  (11-29%) | 420.4 ± 131.1 |
|  | CD163 | 58.32 ± 8.55  (31-75%) | 2153 ± 311.1 |
|  | DC-SIGN | 7.9 ± 0.86  (6-11%) | 1269.8 ± 725.2 |
|  | CD14 | 87.65 ± 3.6  (73-97%) | 8797.2 ± 1267 |
| Intra-  cellular  markers | CD119 | 80.71 ± 5.2  (72-100%) | 46747.3 ± 23730.0 |
|  | iNOS2 | 38.9 ± 10.74  (11-74%) | 2865.8 ± 851.4 |
|  | Arginase I | 65.75 ± 17.58  (40-100%) | 3492.2 ± 1837.3 |
