# Supplementary material for: Replication Rates of Mycobacterium tuberculosis in Human Macrophages Do Not Correlate with Mycobacterial Antibiotic Susceptibility
Source: PLoS One. 2014 Nov 11;9(11):e112426. doi: 10.1371/journal.pone.0112426 (PMC4227709; doi:10.1371/journal.pone.0112426)
Supplement: Table S2 — Antibodies used for macrophage characterization. (DOC) [file pone.0112426.s008.doc]

**Table S2: Antibodies used for macrophage characterization**

| **Antibody** | **Company** | **Product Nr.** | **Dilution** |
| --- | --- | --- | --- |
| mouse anti-human CD206 FITC | BD Pharmingen | #551135 | 1:10 |
| mouse anti-human CD163 PE | BD Pharmingen | #556018 | 1:10 |
| mouse anti-human DC-SIGN PerCP | R&D Systems | #FAB161C | undiluted |
| mouse anti-human CD86 Alexa Fluor 700 | BD Pharmingen | #561124 | 1:40 |
| mouse anti-human CD14 Pacific Blue | Invitrogen | #MHCD1428TR | 1:40 |
| mouse anti-human CD119 | Biolegend | #308602 | 1:65 |
| goat anti-human arginase I | Santa Cruz | (N-20):sc-18351 | 1:100 |
| rabbit anti-human NOS2 | Santa Cruz | (n-20):sc-651 | 1:50 |
| goat anti-mouse Alexa 635 | Invitrogen | A31574 | 1:1000 |
| chicken anti-goat Alexa 488 | Invitrogen | A21467 | 1:1000 |
| donkey anti-rabbit Alexa Fluor 594 | Invitrogen | A21207 | 1:1000 |
